# Humanitarianism as a tool of statecraft: contested authority, sovereign violence, and humanity in the Syrian civil war

**DOI:** 10.1111/disa.12659

**Published:** 2024-09-30

**Authors:** Ümit Seven

**Affiliations:** ^1^ Graduate School of Social Sciences Middle East Technical University Türkiye; ^2^ Center for Security Studies ETH Zürich Switzerland

**Keywords:** civil war, humanitarian aid, sovereignty, statehood, Syria, violence

## Abstract

This research explores the dynamics of interaction between the sovereign state and international humanitarian organisations in alleviating human suffering in the Syrian civil war. Considering civil wars as a rupture in sovereignty, its focus is on the practices of the sovereign state within its social context and the resulting implications for aid organisations. I argue that the Syrian regime has employed state violence, in tandem with administrative and bureaucratic impediments, to reassert its sovereign authority in humanitarian decision‐making processes. This exercise of sovereign power is intertwined with the actions of aid organisations, thereby reshaping power dynamics among the state, aid organisations, and vulnerable populations. Through a qualitative method, I show that the deployment of state violence concomitantly pushes aid organisations, specifically the United Nations, towards enforcing the state sovereignty defined by the regime. As an effect of assertive sovereignty, interpretations of humanitarian principles and practices are continuously negotiated and constructed differently by aid organisations, even though they share a common overarching goal.

## 1 INTRODUCTION

At sunset on 19 September 2016, an airstrike targeted a United Nations (UN) aid convoy and a Syrian Arab Red Crescent (SARC) storage facility in Urum al‐Kubra, located in the western Aleppo Governorate in northwest Syria. The strike resulted in the deaths of at least 14 civilian aid workers. Additionally, the warehouse and an adjacent hospital run by SARC were demolished. Following the incident, the UN halted all aid operations in the country. According to the United Nations Board of Inquiry, the attack was most likely committed by the Syrian Arab Air Force, employing three Syrian Mi‐17 model helicopters and three unspecified fixed‐wing aircraft. The Board also posited that Russia, a staunch ally of Syria and a United Nations Security Council (UNSC) member with veto power, actively participated in the offensive (United Nations Security Council, 2016).

This tragic incident is one of many that starkly exemplify the Syrian regime's^1^ deliberate targeting of aid workers, humanitarian assistance, and civilians during the conflict. Building on this specific instance of violence, this research seeks to understand why aid has been systematically attacked in the Syrian civil war. It examines the articulations of state sovereignty in humanitarian action and their broader implications for aid organisations. When analysing the complex dynamics between international humanitarian actors and the Syrian government in the context of humanitarian action, the central focus lies on the exercise of state sovereignty. This is significant because civil war is an instance of ‘sovereignty rupture’ (Sambanis and Schulhofer‐Wohl, 2019). Central to this inquiry is the role of sovereign power in humanitarian action, where state violence emerges as a strategic tool to generate loyalty, instil fear, and establish legitimacy (Hansen and Stepputat, 2006). This violence is not limited to physical coercion: it also includes symbolic acts and discursive practices that assert authority and control over both individuals and populations (Hansen and Stepputat, 2001).

The aftermath of the Cold War marked a pivotal shift in the interaction between states and humanitarian organisations, leading to extensive scholarly investigation of the subsequent transformations and their consequences. This period has been characterised by a fundamental alteration in conventional concepts of sovereignty, as widely acknowledged in the literature (Falk, 1999; Cohen, 2018). In this evolving context, a substantial body of research has emerged exploring the complex relationships between international aid actors and states. Barnett (2001), for instance, contends that sovereign prerogatives and state pressures have played a pivotal role in shaping the political and pragmatic strategies employed by entities such as the United Nations Refugee Agency within the framework of the international refugee regime. Harvey (2013) similarly observes a trend toward states becoming more assertive in exercising sovereignty in humanitarian action. Governments ultimately hold the prerogative to determine and authorise the presence of humanitarian actors within their sovereign territories. Consequently, Harvey (2013) underlines the need for aid agencies to respect and support better the primary responsibility of states to assist and protect their citizens during disasters, including in conflict situations. De Waal (1997) explores the political dynamics that exacerbated famine and the failures of relief agencies in Sudan, Somalia, and Zaire (now the Democratic Republic of the Congo). He views the ‘internationalization of responsibility for fighting famine’ (de Waal, 1997, p. 217) as a negative trend, because it has bestowed unparalleled dominance and significant practical authority upon institutions that are remote and not unaccountable. While de Waal recognises the political power gained by institutions when taking on an increased role in alleviating human suffering, he does not thoroughly examine how states respond to this trend and the subsequent implications for aid organisations. Similarly, drawing on his professional experience in Médecins Sans Frontières (MSF), Cunningham (2018) suggests that the interaction between international non‐governmental organisations (NGOs) and states is marked by negotiation, with each party pursuing its own goals and interests. His focus is on moral and political imperatives mediated through discourse that shapes the relationship between aid actors and states. Although this account offers a rich understanding of the interaction between international humanitarian NGOs and states, its discursive focus lacks coherence and fails to explain adequately the complexities and drivers of state violence conceptually. Kahn and Cunningham (2013) further assert that the conflict between aid actors and states emerges from the fundamental dilemma that humanitarian action presents for the state's sovereignty and responsibility as the primary provider for its populace, especially in times of crisis. While their arguments appear plausible and reflective of current dynamics, they fall short in addressing the mechanism through which assertive state sovereignty leads to instances of state violence.

Yet, many studies addressing the politicisation of humanitarian assistance predominantly highlight the unintended consequences of aid in armed conflicts. With reference to four cases (Bosnia‐Herzegovina, Kosovo, Rwanda, and Darfur, Sudan), Belloni (2007) contends that coercive actions taken in the name of humanitarian principles have negative ramifications. Fast (2010) underscores the gap between documenting violence against aid workers and explanations of such violence. Narang and Stanton (2017) argue that violence against humanitarian organisations is strategic. They assert that aid organisations become targets in areas where their services strengthen government support. In these studies, a robust theoretical framework to understand comprehensively the violence directed towards aid workers is notably absent.

Within the context of the Syrian civil war, Martínez and Eng (2016) show that UN agencies and most humanitarian organisations contributed to supporting the sovereignty of the Syrian government through their emergency food distributions, despite their pretension to neutrality. Building upon Agamben's (1998) notion of ‘bare life’, they posit that aid actors perpetuate sovereign politics by categorising individuals as those to be saved.

Rebel groups strengthen their control over local populations by using the provision of essential services by aid organisations in the areas they control. As a result, welfare services become political symbols of sovereignty in civil wars. For instance, subsidised bread provision became a key indicator of the state's ability to maintain control and legitimacy during the Syrian civil war (Martínez and Eng, 2017). Against this backdrop, the Syrian regime systematically targeted rebel governance, which challenged its sovereignty by providing services, as part of its strategy to eliminate threats to its authority (Martínez and Eng, 2018). As micro‐governance structures took shape during the civil war, conflicts between authorities emerged, leading to further fragmentation within the country (Abboud, 2016).

Munif (2020) relies on Agamben's (1998) ‘state of exemption’ to explain the politics of life and death in the Syrian civil war, with a focus on micro processes. Meininghaus (2016a) argues that since humanitarian aid is part of the daily life of local communities and the structure of governance during the conflict, unequal distribution of aid between government‐ and opposition‐controlled areas risks the survival of civilians and their future. In another article, Meininghaus (2016b) spotlights the militarisation of medical care in Syria and highlights that by prioritising areas under the regime's control, international aid contributes to the existing power struggles. Kennedy and Michailidou (2017) also show that the World Health Organization's commitment to uphold the sovereignty of the Syrian government hindered its capacity to prevent, detect, and manage the polio outbreak in 2013, as the Bashar al‐Assad regime denied permission for operations in areas controlled by rebels. In a similar vein, Leenders and Mansour (2018) maintain that the Syrian government reinforced its claims to sovereignty by strategically using the international humanitarian system in the context of the Syrian civil war. Wieland (2021) concentrates on a dilemma between international humanitarian norms and political decisions to alleviate human suffering in Syria. Despite the massive violations of international humanitarian law (IHL) and international human rights law, the UN was keen to put an emphasis on the sovereignty of the Syrian government, and this led to a credibility crisis for the world body (Wieland, 2021). In the aftermath of the earthquakes that struck Türkiye and Syria in February 2023, Daher (2023) illustrates how the Syrian regime manipulated humanitarian aid to consolidate its power and pursue normalisation with regional and international actors.

What is lacking in this literature is a focus on the reciprocal relationship between the state and aid organisations in the lead‐up to the provision of humanitarian assistance. Specifically, insufficient attention has been given to the role of state violence as an exercise of sovereign power in these studies, despite the fact that violence is the fundamental aspect of sovereignty (Benjamin, 1978; Hansen and Stepputat, 2006; Vardoulakis, 2013). Against this background, this research explores the contextual conditions of humanitarian action within which the Syrian government employed violence and the consequences of sovereign violence for aid organisations. By examining how sovereign power functions within humanitarian action, this study bridges the gap in analysis of sovereignty, sovereign violence, and international humanitarianism.

As the international community takes on an expanding role in upholding the fundamental principle of humanity within the conflict, the Syrian regime strategically establishes its authority in humanitarian action. In this humanitarian space, violence emerges as a strategic tool employed by the state to uphold the exercise and application of its power. I argue that in response to international efforts and an expanded humanitarian action mandate that impinge upon state sovereignty, the regime's reliance on violence has become a distinct hallmark of the visible articulation of sovereignty authority. This violence stands as the conspicuous embodiment of its sovereign authority. Deliberate acts targeting aid, aid workers, and essential facilities such as bakeries, schools, and hospitals, all constitute a discernible form of political violence. This strategic use of sovereign violence is intrinsically intertwined with the regime's pursuit of preserving its autonomy in times of crisis. This recalibrates the power dynamics that exist between the state, aid organisations, and the vulnerable population with respect to the provision of humanitarian assistance and protection in the conflict. The employment of violence concomitantly pushed aid organisations, specifically the UN, towards enforcing the state sovereignty defined by the regime, which eventually contributed to the authoritarianism in the civil war.

The research is organised as follows. First, I present a theoretical framework that substantiates the inquiry, delving into the social construction of sovereign violence and its contextual formation. This section also explains my methodological approach, encompassing data collection procedures and the application of content analysis techniques. Second, I provide a backdrop for the Syrian civil war and humanitarian action, spotlighting the efforts of international aid actors to alleviate human suffering. While investigating the state of humanitarian assistance in the Syrian civil war, the scope of this study is limited to the time frame between 2011 and 2021. Next, the findings shed light on the ways in which the Syrian government exercises control and exploits humanitarian assistance. It is noteworthy that this section concentrates solely on the Syrian government in relation to humanitarian action, excluding non‐state actors from the scope of this research. Following this, I discuss the Syrian regime's core objectives when deploying violence and imposing restrictions in humanitarian space. The final section (conclusion) underscores the consequences of assertive sovereignty for aid organisations, with a specific focus on the UN.

### 1.1 Sovereignty as a social construct and aid as a site of sovereignty: theoretical and methodological framework

Against the backdrop of multiple meanings and competing definitions of sovereignty, this research conceptualises state sovereignty as a social construction, as expounded by Hansen and Stepputat (2001, 2005, 2006). Expressly, the focus of this work is socially constructed sovereign violence. As a foundational construct, sovereignty is delineated as ‘the notion that there is a final and absolute political authority in the political community. .. and no final and absolute authority exists elsewhere’ (Hinsley, 1986, p. 26). Agamben (1998, p. 16) highlights German political theorist Carl Schmitt's understanding of sovereignty, defined ‘not as the monopoly to sanction or to rule but as the monopoly to decide’. The state's ability to make authoritative decisions is, therefore, the essence of sovereignty (Thomson, 1995). This realist yet tentative definition forms the cornerstone for a constructivist interpretation of sovereignty, which I aim to develop further and apply to our understanding of the Syrian civil war.

In line with constructivist scholarship such as Biersteker and Weber (1996), Hansen and Stepputat (2001, 2005, 2006) conceptualise the territorial state and sovereignty as social constructions. They emphasise two key points: first, sovereignty is a social construct; and second, the exercise of violence is a sovereign act over bodies and populations. As states are subjects in process (Weber, 1998), state behaviours should be understood in relation to institutions composed of social structures. Furthermore, Hansen and Stepputat (2006, p. 295) argue that sovereignty is a ‘tentative and an emergent form of authority grounded in violence’, and that violence is a socially constructed practice of a sovereign state. Therefore, I abandon the notion of sovereignty as an ontological ground of power and instead position state sovereignty within the broader context of its social environment.

As civil war represents a rupture in state sovereignty, articulations of sovereign power become pervasive. Particularly when it comes to the provision of humanitarian assistance in a civil war, state sovereignty claims are a quintessential expression of political power and relational to transnational interactions and organisations that affect the state's authoritative decision‐making process. On a daily basis, state sovereignty is constantly contested, rearranged, and redefined at multiple levels in the Syrian civil war. This has resulted in the fluid contingent forms of order and authority (Martínez and Eng, 2016), and pushed the state to appear in everyday and localised forms (Hansen and Stepputat, 2001).

Given that a territorial entity is expected to demonstrate internal supremacy and external independence to attain sovereignty (Fowler and Bunck, 1996), the Syrian regime, backed up by disciplinary violence, enacts its sovereignty through various means. Its sovereignty claims are constructed in an environment in which the state interacts with the international community and aid organisations. This interaction is multilayered and involves multiple actors, ranging from officials in a village appointed by the Syrian government to senior officials at a higher state level. Through these officials, state sovereignty penetrates all forms of ordinary life in the conflict. In this regard, bureaucratic obstacles and restrictions imposed by the regime should be read as instances of performance in which state sovereignty is at play. Likewise, I consider siege, denial of access, and starvation as a structure of violence linked to the practice of sovereignty and the exercise of power.

Informed by this theoretical foundation, the research pivots its focus to aid professionals, aiming to assess the palpable manifestations of sovereignty. This investigation involves 28 semi‐structured in‐depth interviews with humanitarian professionals who have worked or are currently working for international humanitarian organisations responding to the crisis in the Syrian civil war. Specifically, the interviews were carried out with 12 aid workers from the UN, five from the International Committee of the Red Cross (ICRC), and 11 from different international NGOs, resulting in insights from a total of 16 different aid entities. This design enables me to obtain insights into the operational dynamics of humanitarian assistance, strategies, and tools employed by aid organisations to create humanitarian space, and the regime's response to the international humanitarian efforts in the country. I also explored the participants' thoughts, perceptions, and experiences pertaining to sovereignty and violence in the Syrian civil war. By doing this, I seek to understand the symbolic locus of sovereignty for aid workers in the Syrian civil war.

I employed snowball sampling to access aid workers with various types of aid organisations. Interviewees referred me to their acquaintances. The success of snowball sampling depends on the establishment of trustworthy relationships among potential participants, as highlighted by Cohen and Arieli (2011). I obtained explicit and voluntary verbal consent, which participants had the option to withdraw at any point. The interviews were conducted in English through face‐to‐face meetings in various cities, specifically in Beirut (Lebanon), Gaziantep (Türkiye), and Paris (France), or online, depending on the participant's preference or convenience. Each interview session had a duration ranging from 45–60 minutes and was recorded for subsequent verbatim transcription. The analysis employed a conventional content analysis approach, characterised by an inductive process (Hsieh and Shannon, 2005). This method facilitated the identification of concepts that encapsulated the experiences, perceptions, and thoughts of international humanitarian workers regarding their involvement in humanitarian assistance and their views on state sovereignty in the Syrian civil war. The raw data obtained from the verbatim transcriptions of the interviews served as the foundation for developing themes through a process of further abstraction (Erlingsson and Brysiewicz, 2017).

Through the interviews, I aimed to comprehend the participants' individual trajectories and their experiences and interpretations of their own positions in humanitarian action, particularly contextualising them within sovereign articulations (Soss, 2014). In this respect, in the process of the content analysis of the interviews, the focus was on strategies and tools employed by the Syrian government to enforce its authority and the different aspects of the experiences, thoughts, and perceptions of humanitarian aid workers concerning the provision of humanitarian assistance. Rather than offering a descriptive understanding of humanitarian action in the Syrian civil war, I sought to investigate the practices of humanitarian assistance delivery in relation to the practices of state sovereignty. To this end, through the analysis, I identify state‐imposed challenges as a strategic tool employed to control aid and mobilise resources for its own benefits in humanitarian action. Then, I explore the strategies adopted by aid organisations to navigate these challenges. Following this, I present aid workers' perceptions of and thoughts on state sovereignty and humanitarian norms within the context of the Syrian civil war.

### 1.2 Humanity testing the boundaries of sovereignty

Following the Assad regime's brutal crackdown on protestors, the UN Emergency Relief Coordinator raised concerns about the severe lack of humanitarian access in parts of Syria where violence had intensified in May 2011 (United Nations, 2011). Similarly, the ICRC called for better humanitarian access to people in need, as food, water, and medical supplies ran short in the areas hit by violence in the early days of the uprising (ICRC, 2011). The ICRC's declaration of a ‘non‐international armed conflict’ in June 2012 marked a pivotal moment, triggering the application of IHL (ICRC, 2012). Subsequently, the UN relief agencies and their partners stepped up their humanitarian support and funding to respond to the crisis while calling for unimpeded humanitarian access.

As the conflict transformed into a full‐scale civil war, the humanitarian situation deteriorated rapidly. Thousands of people were displaced internally or left the country in search of safety in neighbouring states. People suffered from a lack of food, fuel, water, electricity, and medical supplies, particularly in Homs, Idlib, Daraa, and Hama (United Nations Human Rights Council, 2012). Most healthcare facilities were destroyed, and 70 per cent of the medical community fled the violence in opposition‐held areas (Coutts and Fouad, 2013).

Aimed at alleviating human suffering in the country, international aid was mainly deployed from the Syrian capital, Damascus, by the ICRC, UN agencies, and a dozen international NGOs (MSF, 2013). In 2015, only 16 international NGOs were officially accredited in Syria, and there were 113 national NGOs authorised to partner with UN agencies. There were also other international and national NGOs operating independently through different modalities, including cross‐border mechanisms, to provide humanitarian assistance to the people in opposition‐held areas.

In response to the mounting engagement of aid organisations in addressing humanitarian concerns during the civil war, the Syrian government sought to assert its influence over these efforts through the deployment of various tools and strategic measures. Its Ministry of Foreign Affairs and Expatriates became the main governmental counterpart of most UN agencies through a memorandum of understanding signed in 2012. SARC was designated as the leading national provider of humanitarian relief and the implementing partner of UN agencies. The organisation also got involved in programme and project designs, particularly in granting permissions to travel, procuring goods and services, and hiring staff (Gorevan, Hemsley, and Sider, 2020). SARC was the first authority to get permission for field missions. Following its endorsement, the request went to the Ministry of Foreign Affairs and Expatriates for approval.

On 22 February 2014, the UNSC unanimously adopted Resolution 2139 to ensure humanitarian access in Syria. While calling on Syrian authorities to allow aid to reach affected people, the resolution articulated the requirement for cross‐line and cross‐border aid delivery to reach people in need. Commenting on the resolution, Assad underscored that it must be implemented ‘with respect for the principles laid out in the UN Charter, international law and the basic foundations of humanitarian work, especially state sovereignty and the role of the state, and principles of neutrality, transparency and non‐politicised assistance’ (France 24, 2014). The first cross‐border operation from Türkiye to northern Syria was conducted by UN agencies in March 2014. Despite the resolution demanding unhindered humanitarian access, aid organisations experienced difficulties in reaching people in need in besieged areas and hard‐to‐reach parts of the country due to fighting. The deteriorating humanitarian situation prompted the UNSC to address this again. On 14 July 2014, it unanimously adopted Resolution 2165 (2014), which authorised the UN and its implementing agencies to deliver cross‐border humanitarian aid through Jordan, Türkiye, and Iraq without the consent of the Syrian government.

Amid the deteriorating humanitarian situation, the Syrian government continued to impose bureaucratic and administrative obstacles to the provision of aid, making delivery more problematic. Especially in government‐controlled areas, the Syrian regime has exerted strict control over the distribution of aid, determining ‘what, where, and to whom’ assistance is allocated and even influencing staff recruitment (Parker, 2013, p. 3). Denying or revoking visas for international humanitarian workers became a tactic of the Syrian government to control aid delivery. Lengthy and cumbersome approval processes for the supply of services, movement of staff, and monitoring and assessment visits adversely affected humanitarian action. The delivery of goods had to be approved by the Ministry of Local Administration and Environment, and certain types of activities, such as legal assistance or training in addressing gender‐based violence, required additional negotiations to gain approval (Gorevan, Hemsley, and Sider, 2020). The delivery of medical supplies to opposition‐held areas was scrutinised particularly. Opposition fighters harassed and detained humanitarian staff at checkpoints and looted humanitarian supplies (Parker, 2013).

As the humanitarian response became part of politics, the UN was accused of breaching humanitarian norms and principles by letting the Assad government control aid deliveries (Hopkins and Beals, 2016). In 2016, 73 aid organisations suspended cooperation with the UN, claiming that the Assad regime exploited relief efforts for its political gains (SAMS, 2016). Later, the Syrian government continued to exploit the delivery of humanitarian assistance to punish its opponents while using humanitarian and reconstruction funding to finance its atrocities (Human Rights Watch, 2019). Aid organisations found themselves in a complex landscape that they had to navigate to help people in need. They often acceded to the demands of the Syrian regime for fear of losing access or being shot down in a sovereign state (Human Rights Watch, 2019).

### 1.3 The machinery of sovereignty and humanity: insights from aid professionals

Concurrent with the research objective, I scrutinise the instruments and strategies employed by the Syrian government in humanitarian action, assessing their alignment with state objectives and their repercussions for humanitarian actors. Simultaneously, I probe into the mechanisms by which humanitarian organisations navigate the impediments and constraints imposed by the government. I also delve into the perceptions, thoughts, and experiences of aid workers concerning state sovereignty and the foundational principles of humanitarian action. Below, I present the qualitative findings of the research structured into the thematic construct.

#### 1.3.1 State‐imposed challenges as sovereign acts in humanitarian action

As the international humanitarian community took steps to meet the humanitarian needs arising from unabated violence in the Syrian civil war on both the global and local level, the Syrian government simultaneously implemented diverse strategies to assert control over aid, aligning with its own political objectives. It imposed numerous obstacles and challenges that hindered humanitarian efforts. Beyond these bureaucratic restrictions, state violence emerged as a primary tool relied upon by the Syrian regime to ‘police its territory and people’ (Thomson, 1995, p. 225). Through these sovereign acts, the Syrian government exercised its sovereign authority in humanitarian action at a time when its existence was under threat. In doing so, it sought to manipulate, exploit, and redirect humanitarian assistance for its own political gains in the civil war.

The Syrian regime arbitrarily denied humanitarian access and imposed political, administrative, and legal barriers to humanitarian organisations. Visa restrictions and deportations became an instrument that it used to punish aid workers and restrict their humanitarian work. While gaining leverage against humanitarian organisations through either withholding or delaying visas, the regime forced aid agencies to comply with its demands. Accordingly, all travel inside the country was also subject to regime approval, and this approval process was also instrumentalised for political ends. Field visit requests were often denied or left without a response, as noted by some study participants:

*We have always faced the challenge of having bureaucratic impediments imposed on humanitarian agents in general and the UN in particular. And this is mainly the issue of visas. So, entering Syria, in principle, is subject to long waiting and vetting processes that, most of the time, end up not granting a visa* (ID23.09).

*[The government] cancelled visas [of international staff] in Syria, just because they communicated with the non‐state armed groups, and that's sensitive* (ID21.09).

The Syrian regime exerted direct influence and pursued a resource mobilisation strategy through various mechanisms in humanitarian action. To operate in Syria, aid organisations were required to partner with local actors that were vetted and approved by the government. Particularly, the UN and international aid organisations had to implement their programmes and projects in direct partnership with SARC and the Syria Trust for Development. Through this mandatory partnership, the Syrian government assigned SARC and the Trust a gatekeeper role. As one interviewee stated:

*The Minister of Foreign Affairs in Damascus has always put the precondition for most of the humanitarian agencies. This includes the fact that you need to work in a partnership with [the] Syrian Arab Red Crescent or Syrian Trust* (ID23.09).

The regime restricted or denied access, particularly to the vulnerable people that it perceived as political opponents. It also redirected aid to its supporters. One interviewee pointed out:

*Because the Syrian government had refused to let any assistance from within Syria, we wouldn't deliver humanitarian assistance to the areas where the opposition groups were mainly located* (ID01.05).

As highlighted by another participant, despite the approval of the regime to deliver assistance, state authorities blocked humanitarian access in the process of delivery:

*If it is a besieged area by the government, they keep us in some of the checkpoints, sometimes for 10 hours, just to make our life difficult. And then maybe they feel that we give up and go back and not deliver* (ID21.09).

Direct violence, hostilities, and targeted attacks against aid workers, as well as arbitrary arrests, substantially impacted on humanitarian work in Syria. The presence of risks in the operating environment forced humanitarians to prioritise their safety and security. One stressed that:

*It's a very dangerous place. We have enormous security risks, which are not only related to the bombardment and shelling, but we also have other risks that expose you, as UN staff, like kidnapping, and etc. … So, it is a dangerous place* (ID19.09).

Another participant working for a UN agency mentioned a propaganda incident that aimed to undermine their humanitarian work:

*The other thing that happened was at one of the vaccination centres. Basically, there was a whole bunch of needles dumped outside one of the vaccination centres. Then, propaganda went up that said ‘you can't trust these people. They're leaving syringes lying around’. But then UNICEF [United Nations Children's Fund] checked to see where those syringes had come from, and they all came from government‐controlled areas. The government had tried to sabotage the campaign by trying to make it up. … We were careless and didn't know what we were doing* (ID05.06).

### 1.4 Strategies and mechanisms used for ensuring humanitarian access

Confronted with a multitude of challenges, aid organisations devised distinctive strategies in alignment with their organisational priorities and humanitarian norms. They utilised a diverse set of tools, strategies, and mechanisms to navigate the complexities arising from the violence, restrictions, and barriers imposed by the Syrian government. The strategies and mechanisms employed by aid organisations are situated at the crossroads of state sovereignty and humanitarian norms and principles.

Aid organisations engaged in humanitarian diplomacy to influence decision‐makers to secure humanitarian access. Each one used different diplomatic tools, strategies, and responses, including advocacy, negotiation, communication, and agreements. These are formal, informal, bilateral, or multilateral, as highlighted in the statement below:

*We negotiated with the Syrian Arab Red Crescent. This was in 2011, 2012. But in 2016, we sent a delegation, an MSF team, to meet with the Minister of Health and the Ministry of Foreign Affairs. But in the end, this was not translated into an operation. They didn't grant us access to the governmental areas because they thought that we were still linked to the opposition* (ID28.04).

This involvement in humanitarian diplomacy occurred across multiple levels. For example, a wide range of practices were undertaken at the international, regional, and local level to reach people in need, formal and informal, as reported by another research participant:

*But you talk with mayors, governors, local people, mukhtar in local communities… they are much more open to talk in a more pragmatic way. So, we have to deal with it at the national, but also very much local level, and also importantly at a technical level* (ID04.06).

Accordingly, aid organisations also applied a range of instruments, encompassing formal and informal negotiations, agreements, and reporting mechanisms. As one interviewee notes, the Norwegian Refugee Council (NRC) was one of the few humanitarian organisations that operated in both government‐controlled and opposition‐held areas, and this was mainly a result of its effective multifaceted humanitarian diplomacy:

*There was a case. The NRC, I remember that it was the only organisation that was able to be on every side. And you know, this would require a lot of diplomacy, a lot of talks, and reporting where you were screened*… (ID09.07).

Aid organisations also established internal rules, operating principles, protocols, and red lines in accordance with their organisational and humanitarian principles that guided their humanitarian action. Nonetheless, by implicitly accepting a political contract, aid agencies made deliberate compromises and trade‐offs to deliver assistance across the country. Such pragmatic trade‐offs require aid workers to focus on benefits rather than costs. Although this, in turn, creates instances that undermine humanitarian norms and principles, it helped humanitarian organisations to secure access elsewhere. In the words of one interviewee:

*Because you do have to compromise somehow, some principles to provide humanitarian assistance. … There is no one answer on where the red line is, and where you start compromising humanitarian principles, it's very much where you put the benchmark* (ID20.09).

Cross‐border humanitarian aid into Syria through Türkiye, authorised by the UNSC, allowed the delivery of lifesaving aid to opposition‐held areas in northwest Syria. The cross‐border aid mechanism became vital in ensuring the flow of assistance into that region, controlled by opposition armed groups. Implementing through local actors and remote management became a preferred modus operandi of aid organisations in the delivery of humanitarian assistance and protection. As highlighted by the study participants, local aid organisations appear to enjoy better humanitarian access in the conflict. In addition, the UN transfers safety and security risks to local aid organisations. One participant remarked:

*National NGOs tend to be the most operational in Syria. They are also the ones that face fewer access constraints. Because they have just better access. They often have people working for them who are from the area, and they have the best relationship with local authorities that you have in Syria these days*… (ID20.09).

Humanitarian actors devised their own strategies and tools to reach vulnerable people in a complex and challenging operational environment. Changing an organisation's name as a disguise or keeping a low profile in the field are among them, as underscored by an interviewee:

*So, some organisations that decided to work in both areas changed their names in the past, like. … Then, they started to work in regime areas. They changed the name of the entity* (ID16.08).

### 1.5 Perceptions, thoughts, and experiences concerning state sovereignty and humanitarian norms and principles

Aid workers' perspectives also offer invaluable insights into the delicate balance between state sovereignty and humanitarian principles in the context of humanitarian action, derived directly from their on‐the‐ground experiences. Through their nuanced reflections, thoughts, and encounters, I unveil the dynamic interaction between sovereignty and humanitarian norms. I demonstrate how aid workers themselves perceive their role and authority in the Syrian civil war. What becomes apparent from these findings is that, despite the shared overarching goal, aid workers employed by diverse organisations have contrasting experiences and beliefs regarding state sovereignty and humanitarian norms and principles.

Some participants stated that the Syrian regime's sovereignty should be recognised and respected in the conflict. They underlined that the state's permission is required for aid organisations to operate in the country. Specifically, one participant working for the UN referred to General Assembly Resolution 46/182, adopted in 1991, which created the humanitarian system, to highlight the UN's obligations in the country:

*We, as OCHA [UN Office for the Coordination of Humanitarian Affairs], and most humanitarian organisations, are guided by General Assembly Resolution 46/182. And based on that, we need to respect the sovereignty of the state. For that sake, we can only operate when we have a Security Council resolution. Especially when it's a cross‐border operation. We respect the sovereignty and territorial integrity of the state* (ID23.09).

Administrative and legal barriers in humanitarian action became an instrument that the Syrian government employed to show its power, as noted by another participant:

*I think because the Syrian regime wanted to show that they were in charge. They would say where people can go… they just really restrict access to non‐regime areas* (ID01.05).

Most of the interviewees reported that humanitarian norms and principles were violated by aid organisations in the provision of humanitarian assistance in the Syrian conflict. In particular, they emphasised the limits of the UN as an intergovernmental organisation mandated to uphold the principle of non‐interference in the domestic affairs of a member state. Participants also expressed negative feelings and thoughts on the role of the UN in the provision of humanitarian assistance and protection to vulnerable populations. Furthermore, beyond the role of the UN, few participants thought that the international community was ineffective in principled aid delivery in the conflict. One said:

*Because the UN is invited to the scene and the reality is that they [UN] don't have to be there. Therefore, the government's going to request them to do certain things* (ID01.05).

Aid organisations grappled with challenging decisions regarding where and whom to assist, given the regime's interference in aid delivery. These circumstances presented difficulties in upholding humanitarian norms and principles. One participant highlighted this dilemma:

*They grant access to the areas; they give you permission to work, and they can control so many things… and I think it's very difficult to say that humanitarian aid is neutral because, at the end of the day, you stand with a choice between helping people and being okay with that, or saying no to influence and then saying no to helping people* (ID22.09).

Yet, despite the complexity on the ground and challenges, participants underlined the significance of adhering to humanitarian norms and principles in humanitarian action. One put it as follows:

*I think that humanitarian principles should guide the way you interact first with the government and second with your partners* (ID26.10).

While upholding the supremacy of IHL over sovereignty, one participant working for an international NGO stated that humanitarian work should not be overambitious:

*I think it [IHL] should override it [state sovereignty]. But it doesn't. You know, this is why governments can stop you coming to [their country], going and doing whatever they like basically. But I don't think it should be that way. I think humanitarian work needs to be, you know, back to the basics* (ID01.05).

The Syrian government, as the ultimate governing body in the country, enforced a distinct set of regulations in the distribution of humanitarian aid, constituting a blatant breach of IHL. Participants highlighted its reliance on territorial control as a means of exercising sovereign authority to enforce its own regulations. As per their perspectives, in areas held by the opposition, where the Syrian government lacked control, participants argued that the regime forfeits its sovereign privileges. Thus, in their perspective, humanitarian norms and principles take precedence over state sovereignty.

## 2 THE TRIUMPH OF STATE SOVEREIGNTY IN HUMANITARIAN ACTION

Aid organisations operating within the country, alongside the international community at the global level, infringed upon sovereign state functions by undertaking actions in the name of humanity to alleviate human suffering in the civil war. For instance, by categorising people as vulnerable and entitled to receive aid or essential services, aid organisations assumed a sovereign‐like role. Moreover, their operations in areas controlled by non‐state armed groups had the potential to accord status, recognition, and legitimacy to armed groups, as highlighted by the study participants. Striking a balance between delivering humanitarian assistance and the apprehension of inadvertently endorsing an opposition faction posed an ongoing challenge to aid organisations. Furthermore, in various instances, humanitarian aid faced theft and diversion by armed groups, redirecting resources to advance their own interests and political objectives.

In response to the outgrowing role of aid organisations in the conflict, the Syrian regime employed various tools to safeguard its sovereignty and maintain control over its territory. The government's strategy was inherently multifaceted. It imposed political, administrative, and physical barriers to humanitarian action, adopting a sovereignty‐first narrative. Rather than being random, these obstacles and restrictions, as the findings show, are part of a strategy that aims to uphold and prioritise state sovereignty in the conflict. In other words, the Syrian government, as the exclusive authority in the country, firmly controlled the decision‐making process relating to humanitarian action, often disregarding IHL. It set the conditions for aid organisations willing to operate in the country to benefit from humanitarian assistance. This assertive approach prioritised its authority over compliance with IHL. The state's actions were shaped and maintained in response to the social environment, particularly in consideration of the reactions and responses of the international humanitarian community both at the international and local level.

The findings of this research reveal the Syrian regime's core objectives when imposing restrictions and deploying violence in spaces of contested authority. First, the Syrian government sought to project itself as the only authority in the humanitarian decision‐making process. It rejected projects offered by international organisations, restricted the movements of aid workers, or diverted aid and funding. In doing so, it leveraged itself against aid organisations, showing its supreme power. Second, the Syrian government exercised its sovereignty over people in spaces of contested authority, rather than over things, as highlighted by Hansen and Stepputat (2005). By exerting control over and through the manipulation of aid, it instrumentalised humanitarian assistance to penalise civilian populations that it perceived as opponents while rewarding those it deemed loyal. This instrumentalisation of aid became part of a broader strategy in which violence emerged as an effective tool that the Syrian regime utilised in its sovereignty claims.

However, despite facing international condemnation and various reactions, the Syrian regime persisted in employing indiscriminate violence against civilians and violating human rights throughout the conflict. It sought to legitimise its actions by invoking the principles of safeguarding its sovereignty and territorial integrity. Additionally, on the international stage, Syria's close ally, Russia, served as a staunch defender of the regime. In response to any unilateral or bilateral actions targeting the Syrian regime, Russia and China, as veto‐wielding members of the UNSC, consistently denounced such actions as ‘aggression against a sovereign state’ in violation of international law. In line with the findings, this aspect raises the question of whether being backed by a superpower serves as an incentive to push the boundaries when it comes to deploying violence that flagrantly violates IHL. Further research is necessary, therefore, to understand comprehensively the circumstances in which states may deploy violence and violate IHL.

The findings show that the practices and sovereign acts via which the Syrian government aims to rule, regulate, and control international humanitarian aid form a strategic interaction with international humanitarian organisations. These state practices encompass the complex intertwining of structural, symbolic, and direct forms of violence. Beyond the direct violence directed at humanitarian aid and aid workers, the study participants underscored the prevalence of numerous types of violence and the significance in their interactions with the Syrian government. The findings support Hansen and Stepputat's (2001) argument that violence is not limited to over‐physical acts but can also entail subtle, everyday forms. This spatial violence, whether physical or symbolic, is deeply entwined with social relations and power structures. This multiplicity of violence has inherently influenced the way in which aid organisations have operated within the context of the Syrian civil war.

These assertive articulations of state sovereignty have had notable implications for the activities of aid organisations in the civil war. Unlike a unified and consistent approach, the global governance of humanity takes varied forms depending on the actors involved. The impact of assertive sovereignty becomes evident as the meanings and practices linked to humanitarian assistance continually undergo negotiation and reinterpretation within relief organisations, despite their shared overarching objective. Consequently, this research emphasises the distinctions among different types of aid organisations operating in the Syrian civil war. Specifically, professionals associated with the UN exhibit a stronger commitment, in comparison to their counterparts at the ICRC and local and international NGOs, to the importance of upholding and acknowledging state sovereignty within the intricate environment of the civil war. The UN attaches importance to respecting state sovereignty as it strives to ensure the safety of its operations and uphold humanitarian access across the entire country. It concedes to regime demands out of concern over losing access. It is noteworthy that the UN's unwavering dedication to upholding the principles of state sovereignty distinguishes it from both local and international NGOs.

## 3 CONCLUSION

Expanding upon Hansen and Stepputat's (2005, 2006) assertion that sovereignty is constructed and wielded through spectacular violence, this research has shed light on the intricate intersection of sovereignty and humanitarian action within the Syrian civil war. Humanitarian space emerges as a locus of sovereignty, not merely as a passive backdrop, but as a dynamic arena shaped by physical, legal, and social factors. This dynamic exemplifies the intricate relationship between the state and aid organisations, continually influenced by and reshaped through their interactions. States, as central wielders of power, exercise their sovereignty by moulding and governing the spaces within their borders. The regulation of space is thus an integral aspect of state sovereignty. It is within this context that the Syrian regime, by manipulating and redirecting aid, imposing restrictions on humanitarian operations, and resorting to violence, aimed to assert its sovereign authority in the humanitarian space. Considering civil wars as disruptions of state sovereignty, I have underscored the Syrian regime's deliberate use of violence and bureaucratic obstacles as a strategic tool to fortify its sovereignty. In this regard, this study provides further evidence of the strategic use of sovereignty claims, aligning with previous literature such as Martínez and Eng (2016), Kennedy and Michailidou (2017), Leenders and Mansour (2018), and Daher (2023). Beyond that, it advances this research field by offering a framework to interpret violence as a sovereign act and to analyse its implications for humanitarian action.

While contextualising the emergence of sovereign violence, my focus was on the interaction between the Syrian government and aid organisations in the civil war. Owing to their very nature, aid organisations strove to uphold international humanitarian norms in their efforts to alleviate suffering. In a setting in which aid organisations assume an extended role in the name of humanity, the Syrian government sought to assert its sovereignty by making authoritative decisions that directly affect its very existence. These assertive sovereign acts, including sovereign violence and bureaucratic obstacles, took shape in response to the international community's efforts and actions at both the global and local level to assuage human suffering in the Syrian civil war. Hence, the creation of a humanitarian space has been shaped by its surrounding social, political, and legal circumstances, making it a contingent outcome. At the intersection of state sovereignty and humanitarian principles, there exists a power struggle that determines the boundaries of humanitarian space. This sovereign violence, therefore, should not be viewed as an ontological source of power, but rather as a relational concept that takes shape within the broader context of its social surroundings.

It is worth noting that not only did direct state violence contribute to the shaping of power dynamics, but also sovereign actions, such as visa restrictions, humanitarian access denials, and administrative and bureaucratic obstacles, significantly impacted on humanitarian operations in the Syrian civil war. These sovereign acts in turn compelled aid organisations, specifically the UN in contrast to the ICRC and international NGOs, towards enforcing the state sovereignty defined by the regime. In this process, humanitarian norms and principles took a subordinate role to state sovereignty. Intentional compromises and trade‐offs often surfaced as a strategic measure that aid organisations employed to maintain the continuity and safety of their operations, even when this entailed a departure from humanitarian norms and principles.

The findings of the research suggest that administrative obstacles and state violence are intricately linked to international humanitarian efforts, working in tandem to bolster state authority during the Syrian civil war. Consequently, it is essential for aid organisations to recognise the effect of their humanitarian interventions on power dynamics and the resulting responses of the state. This underscores the importance of sensitive programming, cohesive collaboration, and united action among aid organisations, rather than operating in isolation. Moreover, aid organisations must craft strategies that carefully balance adherence to humanitarian principles with ensuring access to those in need. Such a nuanced approach enables them to navigate the complexities of operational landscapes more effectively.

## DISCLOSURE STATEMENT

No potential conflict of interest was reported by the author(s).

## FUNDING STATEMENT

The author did not receive support from any organisation for the submitted work.

## ENDNOTE

^1^
Although often treated as synonymous, the terms ‘regime’ and ‘government’ carry distinct meanings. As Lawson (1993, p. 185) emphasises, ‘governments may come and go, while the regime remains more or less in place’. Therefore, when I mention ‘the Syrian regime’, I am referring to the government of Syria, but specifically highlighting its oppressive and authoritarian nature, which leads to the suppression of political dissent.

## References

[disa12659-bib-0001] Abboud, S.N. (2016) ‘Conflict, governance, and decentralized authority in Syria’. In M. Beck , D. Jung , and P. Seeberg (eds.) The Levant in Turmoil: Syria, Palestine, and the Transformation of Middle Eastern Politics. Palgrave Macmillan, New York City, NY. pp. 57–77.

[disa12659-bib-0002] Agamben, G. (1998) Homo Sacer: Sovereign Power and Bare Life. Stanford University Press, Stanford, CA.

[disa12659-bib-0003] Barnett, M. (2001) ‘Humanitarianism with a sovereign face: UNHCR in the global undertow’. The International Migration Review. 35(1). pp. 244–277.

[disa12659-bib-0004] Belloni, R. (2007) ‘The trouble with humanitarianism’. Review of International Studies. 33(3). pp. 451–474.

[disa12659-bib-0005] Benjamin, W. (1978) Reflections: Essays, Aphorisms, Autobiographical Writings. Harcourt Brace Jovanovich, New York City, NY.

[disa12659-bib-0006] Biersteker, T.J. and C. Weber (1996) ‘The social construction of state sovereignty’. In C. Weber and T.J. Biersteker (eds.) State Sovereignty as Social Construct. Cambridge University Press, Cambridge. pp. 1–21.

[disa12659-bib-0007] Cohen, N. and T. Arieli (2011) ‘Field research in conflict environments: methodological challenges and snowball sampling’. Journal of Peace Research. 48(4). pp. 423–435.

[disa12659-bib-0008] Cohen, R. (2018) ‘Humanitarian imperatives are transforming sovereignty’. Brookings Institution website. 1 January. https://www.brookings.edu/articles/humanitarian-imperatives-are-transforming-sovereignty/ (last accessed on 9 September 2024).

[disa12659-bib-0009] Coutts, A. and F.M. Fouad (2013) ‘Response to Syria's health crisis—poor and uncoordinated’. The Lancet. 381(9885). pp. 2242–2243. 10.1016/s0140-6736(13)61421-x 23819155

[disa12659-bib-0010] Cunningham, A.J. (2018) International Humanitarian NGOs and State Relations: Politics, Principles, and Identity. First edition. Routledge, London.

[disa12659-bib-0011] Daher, J . (2023) *The Aftermath of Earthquakes in Syria: The Regime's Political Instrumentalisation of a Crisis*. Research Project Report: Syrian Trajectories Project. Issue 2023/04. 27 February. European University Institute, San Domenico di Fiesole.

[disa12659-bib-0012] de Waal, A. (1997) Famine Crimes: Politics & the Disaster Relief Industry in Africa. James Currey, Oxford.

[disa12659-bib-0013] Erlingsson, C. and P. Brysiewicz (2017) ‘A hands‐on guide to doing content analysis’. African Journal of Emergency Medicine. 7(3). pp. 93–99. 30456117 10.1016/j.afjem.2017.08.001 PMC6234169

[disa12659-bib-0014] Falk, R. (1999) ‘Human rights, humanitarian assistance and the sovereignty of states’. In K.M. Cahill (ed.) A Framework for Survival: Health, Human Rights, and Humanitarian Assistance in Conflicts and Disasters. Routledge, London. pp. 122–136.

[disa12659-bib-0015] Fast, L. (2010) ‘Mind the gap: documenting and explaining violence against aid workers’. European Journal of International Relations. 16(3). pp. 365–389.

[disa12659-bib-0016] Fowler, M.R. and J.M. Bunck (1996) ‘What constitutes the sovereign state?’. Review of International Studies. 22(4). pp. 381–404.

[disa12659-bib-0017] France 24 (2014) ‘Syria agrees to UN aid resolution if “state sovereignty” respected’. Website. 22 February. https://www.france24.com/en/20140222-syria-un-security-council-unanimously-humanitarian-resolution (last accessed on 9 September 2024).

[disa12659-bib-0018] Gorevan, D. , M. Hemsley , and R. Sider (2020) Hard Lessons: Delivering Assistance in Government‐Held Areas of Syria. Joint Agency Briefing Paper. July. Oxfam GB, Oxford.

[disa12659-bib-0019] Hansen, T.B. and F. Stepputat (2001) ‘Introduction: states of imagination’. In T.B. Hansen and F. Stepputat (eds.) States of Imagination: Ethnographic Explorations of the Postcolonial State. Duke University Press, Durham, NC. pp. 1–38.

[disa12659-bib-0020] Hansen, T.B. and F. Stepputat (2005) ‘Introduction’. In T.B. Hansen and F. Stepputat (eds.) Sovereign Bodies: Citizens, Migrants, and States in the Postcolonial World. Princeton University Press, Princeton, NJ. pp. 1–36.

[disa12659-bib-0021] Hansen, T.B. and F. Stepputat (2006) ‘Sovereignty revisited’. Annual Review of Anthropology. 35(1). pp. 295–315.

[disa12659-bib-0022] Harvey, P. (2013) ‘International humanitarian actors and governments in areas of conflict: challenges, obligations, and opportunities’. Disasters. 37(S2). pp. S151–S170. 23876011 10.1111/disa.12019

[disa12659-bib-0023] Hinsley, F.H. (1986) Sovereignty. Second edition. Cambridge University Press, Cambridge.

[disa12659-bib-0024] Hopkins, N. and E. Beals (2016) ‘How Assad regime controls UN aid intended for Syria's children’. *The Guardian* website. 29 August. https://www.theguardian.com/world/2016/aug/29/how-assad-regime-controls-un-aid-intended-for-syrias-children (last accessed on 9 September 2024).

[disa12659-bib-0025] Hsieh, H‐F . and S.E. Shannon (2005) ‘Three approaches to qualitative content analysis’. Qualitative Health Research. 15(9). pp. 1277–1288. 16204405 10.1177/1049732305276687

[disa12659-bib-0026] Human Rights Watch (2019) Rigging the System: Government Policies Co‐Opt Aid and Reconstruction Funding in Syria. Human Rights Watch, New York City, NY.

[disa12659-bib-0027] ICRC (International Committee of the Red Cross) (2011) ‘Syria: request for immediate access to violence‐stricken areas’. ReliefWeb website. 10 June. https://reliefweb.int/report/syrian-arab-republic/request-immediate-access-violence-stricken-areas (last accessed on 9 September 2024).

[disa12659-bib-0028] ICRC (2012) ‘Syria: ICRC and Syrian Arab Red Crescent maintain aid effort amid increased fighting’. ReliefWeb website. 17 July. https://reliefweb.int/report/syrian-arab-republic/icrc-and-syrian-arab-red-crescent-maintain-aid-effort-amid-increased (last accessed on 9 September 2024).

[disa12659-bib-0029] Kahn, C. and A. Cunningham (2013) ‘Introduction to the issue of state sovereignty and humanitarian action’. Disasters. 37(S2). pp. S139–S150. 23875977 10.1111/disa.12018

[disa12659-bib-0030] Kennedy, J. and D. Michailidou (2017) ‘Civil war, contested sovereignty and the limits of global health partnerships: a case study of the Syrian polio outbreak in 2013’. Health Policy and Planning. 32(5). pp. 690–698. 28453719 10.1093/heapol/czw148

[disa12659-bib-0031] Lawson, S. (1993) ‘Conceptual issues in the comparative study of regime change and democratization’. Comparative Politics. 25(2). pp. 183–205.

[disa12659-bib-0032] Leenders, R. and K. Mansour (2018) ‘Humanitarianism, state sovereignty, and authoritarian regime maintenance in the Syrian war’. Political Science Quarterly. 133(2). pp. 225–257.

[disa12659-bib-0033] Martínez, J.C. and B. Eng (2016) ‘The unintended consequences of emergency food aid: neutrality, sovereignty and politics in the Syrian civil war, 2012–15’. International Affairs. 92(1). pp. 153–173.

[disa12659-bib-0034] Martínez, J.C. and B. Eng (2017) ‘Struggling to perform the state: the politics of bread in the Syrian civil war’. International Political Sociology. 11(2). pp. 130–147. 33381222 10.1093/ips/olw026 PMC7743994

[disa12659-bib-0035] Martínez, J.C. and B. Eng (2018) ‘Stifling stateness: the Assad regime's campaign against rebel governance’. Security Dialogue. 49(4). pp. 235–253. 30034121 10.1177/0967010618768622 PMC6041736

[disa12659-bib-0036] Meininghaus, E. (2016a) ‘Humanitarianism in intra‐state conflict: aid inequality and local governance in government‐ and opposition‐controlled areas in the Syrian war’. Third World Quarterly. 37(8). pp. 1454–1482.

[disa12659-bib-0037] Meininghaus, E. (2016b) ‘Emergency aid in intra‐state war and implications for post‐conflict reconstruction: the Syrian medical system’. Contemporary Levant. 1(2). pp. 108–124.

[disa12659-bib-0038] MSF (Médecins Sans Frontières) (2013) Syria Two Years On: The Failure of International Aid So Far. Press Dossier. March. MSF, Geneva.

[disa12659-bib-0039] Munif, Y. (2020) The Syrian Revolution: Between the Politics of Life and the Geopolitics of Death. Pluto Press, London.

[disa12659-bib-0040] Narang, N. and J.A. Stanton (2017) ‘A strategic logic of attacking aid workers: evidence from violence in Afghanistan’. International Studies Quarterly. 61(1). pp. 38–51.

[disa12659-bib-0041] Parker, B. (2013) ‘Humanitarianism besieged’. *Humanitarian Exchange*. 59 (November). Humanitarian Practice Network, Overseas Development Institute, London. pp. 3–5.

[disa12659-bib-0042] Sambanis, N. and J. Schulhofer‐Wohl (2019) ‘Sovereignty rupture as a central concept in quantitative measures of civil war’. Journal of Conflict Resolution. 63(6). pp. 1542–1578.

[disa12659-bib-0043] SAMS (Syrian American Medical Society) (2016) ‘Letter to the UN’. Position Paper. 9 September. https://www.sams-usa.net/wp-content/uploads/2016/09/UN-Position-Paper-final-2.pdf (last accessed on 11 September 2024).

[disa12659-bib-0044] Soss, J. (2014) ‘Talking our way to meaningful explanations: a practice‐centered view of interviewing for interpretive research’. In D. Yanow and P. Schwartz‐Shea (eds.) Interpretation and Method: Empirical Research Methods and the Interpretive Turn. Routledge, London. pp. 161–182.

[disa12659-bib-0045] Thomson, J.E. (1995) ‘State sovereignty in international relations: bridging the gap between theory and empirical research’. International Studies Quarterly. 39(2). pp. 213–233.

[disa12659-bib-0046] United Nations (2011) ‘Syria: UN official voices concern about lack of humanitarian access’. UN News website. 10 May. https://news.un.org/en/story/2011/05/374602 (last accessed on 9 September 2024).

[disa12659-bib-0047] United Nations Human Rights Council (2012) Report of the Independent International Commission of Inquiry on the Syrian Arab Republic . A/HRC/19/69. 22 February. https://digitallibrary.un.org/record/721894?ln=en&v=pdf (last accessed on 9 September 2024).

[disa12659-bib-0048] United Nations Security Council (2016) Letter dated 21 December 2016 from the Secretary‐General addressed to the President of the Security Council . S/2016/1093. 21 December. https://digitallibrary.un.org/record/853646?ln=en&v=pdf (last accessed on 9 September 2024).

[disa12659-bib-0049] Vardoulakis, D. (2013) Sovereignty and Its Other: Toward the Dejustification of Violence. Fordham University Press, New York City, NY.

[disa12659-bib-0050] Weber, C. (1998) ‘Performative states’. Millennium. 27(1). pp. 77–95.

[disa12659-bib-0051] Wieland, C. (2021) Syria and the Neutrality Trap: The Dilemmas of Delivering Humanitarian Aid Through Violent Regimes. Bloomsbury Publishing, London.

